# Acute urinary retention in benign prostatic hyperplasia: Risk factors and current management

**DOI:** 10.4103/0970-1591.35050

**Published:** 2007

**Authors:** K. Muruganandham, Deepak Dubey, Rakesh Kapoor

**Affiliations:** Department of Urology and Renal Transplantation, Sanjay Gandhi Postgraduate Institute of Medical Sciences, Lucknow, India

**Keywords:** Acute urinary retention, benign prostatic hyperplasia

## Abstract

Acute urinary retention (AUR) is one of the most significant, uncomfortable and inconvenient event in the natural history of benign prostatic hyperplasia (BPH). The immediate treatment is bladder decompression using urethral or suprapubic catheterization. Several factors have been identified that are associated with or precipitate AUR. It is useful to classify AUR as BPH-related or not, than spontaneous or precipitated when the initial management is considered. Use of prophylactic 5 a-reductase inhibitors can prevent AUR in men with BPH having moderate to severe lower urinary tract symptoms and large prostate size. Alpha blockers can prevent AUR in symptomatic BPH patients and also facilitate catheter removal following episodes of spontaneous AUR. Anticholinergics can be safely combined with alpha blockers in symptomatic BPH patients without increasing the risk of AUR. Surgical treatment carries a higher rate of morbidity and mortality in men presenting with AUR compared to those presenting with symptoms alone. Urgent prostatic surgery after AUR is associated with greater morbidity and mortality than delayed prostatectomy. Alpha blockers mainly help to delay the surgery and may avoid surgery altogether in a subgroup of patients. TURP remains the “gold standard” if a trial without catheter fails. Alternative minimally invasive procedures can be considered in poor-risk patients, but its value is yet to be established.

Acute urinary retention (AUR) represents one of most significant and painful events in the natural history of benign prostatic hyperplasia (BPH). Up to a third of patients undergoing surgical treatment for BPH present with acute urinary retention (AUR).[1] Acute urinary retention is associated with significant anxiety, discomfort and patient inconvenience. The impact on patients' health-related quality of life is comparable to an attack of renal colic.[2] Acute urinary retention was once considered an absolute indication for prostatectomy but the patients' desire to avoid surgery and development of successful medical management has led to a more conservative approach commonly being adopted. The approach and management of AUR has undergone a profound change over the last decade. We herein discuss the risk factors and recent trends in the management of AUR secondary to BPH [Figure 1].

**Figure 1:**
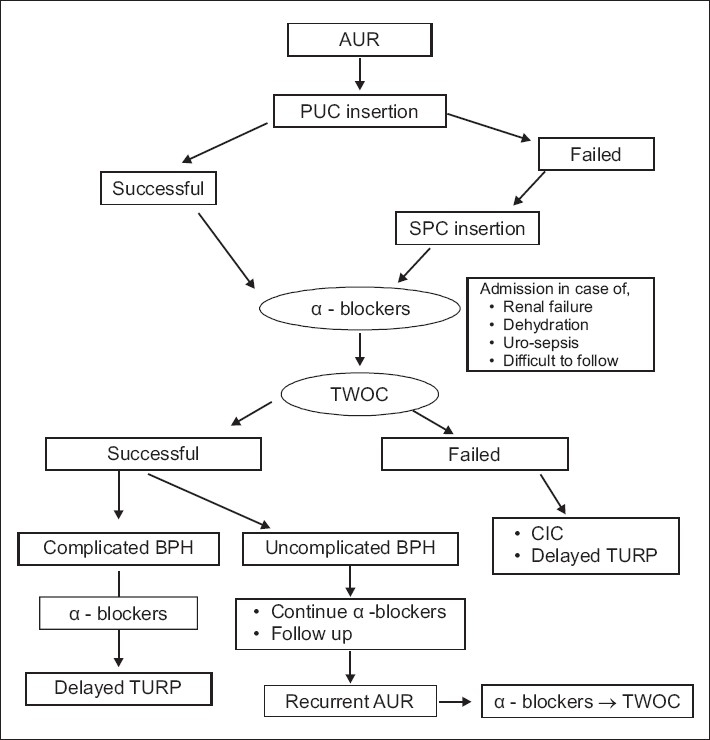
Flow chart for the management of AUR in BPH

## EPIDEMIOLOGY

Ten per cent of men in their seventies and 30% in their eighties will have AUR within the next five years.[3] Benign prostatic hyperplasia is the cause for the AUR in at least 65% of men presenting with AUR.[4] Men with AUR often have lower urinary tract symptoms (LUTS) for an average of 32 months prior to the AUR.[5] The best data from men diagnosed with BPH stem from the Proscar Long-Term Efficacy and Safety Study (PLESS). In PLESS, out of 1,376 placebo-treated men with enlarged prostates and moderate symptoms who had complete follow-up over four years; 99 of them experienced an episode of AUR, for a calculated incidence rate of 18/1000 person-years.[6] Acute urinary retention is the presenting feature in 24-42% of men undergoing prostatectomy.[1] In the Olmsted county study of the natural history of BPH, the risk of AUR was 1.6% at five years for men aged 40-49 years and 10% at 70-79 years.[3] The risk of recurrent AUR in men who presented with first episode of AUR has been reported as 76-83% in men with diagnosed BPH without the use of alpha blockers.[7]

Cathcart *et al*. reported from a large database of 165,527 men who presented with AUR, how the incidence of primary and recurrent AUR changed in England between 1998 and 2003. The incidence of primary AUR was 3.06/1,000 men yearly. The incidence of AUR decreased from 3.17/1,000 men yearly in 1998 to 2.96/1,000 men yearly in 2003. Surgical treatment following spontaneous AUR decreased from 32% in 1998 to 26% in 2003. This trend coincided with a 20% increase in the rate of recurrent AUR. The slight decrease in the incidence of primary AUR suggests that the shift away from surgical treatment of BPH has not resulted in an increase in the AUR. The increase in recurrent AUR suggests that the observed decrease in surgery after AUR might have put more men at risk for AUR recurrence.[8]

## NATURAL HISTORY OF AUR

Men who experience an episode of AUR are at higher risk for subsequent episodes of AUR. The likelihood of a second episode of AUR following a Trial without catheter (TWOC) ranges from 38-56%. It depends on the post void residual urine (PVR), prostate size and the time interval between catheter insertion and the TWOC.[9] Klarskov *et al*. reported that at one year, 85% of men had undergone surgical intervention following an episode of AUR.[10] Because of the risk of a second episode of AUR, many men will elect to proceed with surgical intervention. Emberton M *et al*. analyzed data from 5,792 men with BPH-related symptoms on medical management and reported that prior AUR is a strong predictor of recurrent AUR with a hazard ratio of 3.75% (95% CI 1.58 to 8.89).[11]

## ETIOPATHOGENESIS

Broadly, the causes of AUR can be classified into three categories; any event which increases the resistance to the flow of urine, interruption of bladder innervation and any situation which causes the bladder to over-distend. The precise mechanism which precipitates AUR in BPH has not been fully described. The following factors were thought to be responsible for the occurrence of AUR in BPH.

### Prostatic inflammation

Prostatic inflammation may be an important predictor of disease progression, especially the first occurrence of AUR. Tuncel *et al*. reported a higher incidence of prostatic inflammation in men with AUR than in those having LUTS only (54.7% vs. 28.9%).[12] In the MTOPS study, of 1,197 patients who underwent baseline prostate biopsies, 544 were found to have acute inflammatory changes and 513 had chronic inflammatory changes. Patients with inflammation had larger prostate glands and a higher PSA (both accepted as risk factors for AUR) than those without inflammation and were more likely to progress symptomatically or to undergo surgery. Within the placebo group also, all the patients who developed AUR had inflammation in their baseline biopsies.[13] Almost all BPH specimens show inflammatory infiltrates at histologic examination, but correlation to bacterial or other foreign antigens has not been established. Recognition of prostate secretion products by autoreactive T cells and animal models on experimental prostatitis demonstrates an autoimmune component to chronic inflammation. The infiltrate consists predominantly of chronically activated CD4(+) T lymphocytes, which are permanently recruited to the prostate tissue via elevated expression of interleukin 15 (IL-15) and interferon gamma (IFN-gamma), pro-inflammatory cytokines produced by smooth muscle and T cells, respectively.[37]

### Prostatic infarction

Spiro and colleagues found evidence of infarction in 85% of prostates removed for AUR versus only 3% in prostates of men having surgery for LUTS.[14] Prostatic infarction could result from distortion of the intraglandular vascular supply, trauma (including instrumentation) and infection. The exact mechanism of prostatic infarction leading to AUR has not been determined. There is now a hypothesis whereby prostatic infarction leads to swelling and a rise in intraprostatic urethral pressure (in the peri-ischemic zone), which in turn stimulates efferent alpha-adrenergic nerves resulting in further rise of intraurethral pressure ultimately resulting in AUR.[15] However, in another study Anjum and colleagues have documented similar rates of infarction in 35 men with and without AUR (1.9% vs. 3.0%). Hence the role of prostatic infarction in the pathogenesis of AUR is still controversial.[16]

### Stromal/Epithelial ratio

In a prospective controlled clinico-pathological study comparing 35 patients who presented with AUR secondary to BPH and 35 patients with only refractory LUTS, those presenting with AUR had a significantly higher epithelial component (71% vs. 60%) than patients with LUTS only. Differential epithelial/stromal growth may predispose to infarction through effects on the vascular supply which may result in AUR. This may help to explain why finasteride, which mainly acts on epithelial tissue, can decrease the incidence of AUR.

### Constipation and AUR

It is commonly thought that constipation will precipitate an episode of urinary retention, whilst published evidence that this occurs in adults in the absence of bladder outlet obstruction, neurological disease or fecal impaction is scarce. In the Alfuzosin in Acute Urinary Retention (ALFAUR) study a careful history of bowel habits was taken from 363 men presenting with AUR related to BPH. A history of constipation did not influence whether or not a trial of voiding was successful. Therefore a history of constipation, as opposed to fecal impaction, should not affect how AUR is to be managed. But it may provide an indicator of how the patient will fare in the longer term in terms of need for surgery and recurrent AUR.

### Postoperative AUR

It is accepted that anesthesia, analgesia, intravenous fluids and pain may lead to the development of postoperative AUR. In a study by Hawa *et al*., AUR was found to be associated with increasing age and an intravenous infusion > 750 ml. The combination of suppressed consciousness and fluid infusion lead to the development of AUR. Postoperative AUR occurs whether or not BPH is present.[17]

### Genitourinary instrumentation

Acute urinary retention may develop following genitourinary diagnostic procedures, such as cystoscopy, biopsy of the prostate and ureteroscopy. Rigid cystoscopy precipitates AUR by causing irritation to lower genitourinary tissues and hematuria. Flexible cystoscopy has now replaced rigid cystoscopy, it causes less trauma and irritation and fewer cases of AUR. Prostatic biopsies performed using large Tru-cut needles under digital control result in significant trauma to the prostate leading to swelling, hematoma and hematuria which may in turn result in AUR. When prostatic biopsies are performed under TRUS guidance using much smaller biopsy needles AUR is extremely rare.[18] Acute urinary retention may develop following ureteroscopy also, because the procedure is performed using a general anesthetic and causes bladder irritation.

### Cerebrovascular accident (CVA) and alcohol

The neurological impact of a stroke is associated with impaired bladder emptying which may manifest as AUR. Anecdotal evidence supports that alcohol ingestion can precipitate AUR. The mechanisms involved are likely to be a combination of neurological suppression and fluid overload. Alcohol ingestion and CVA may lead to AUR in the presence or absence of BPH.

### Urinary tract infection (UTI)

Urinary tract infection in the male may complicate or precipitate AUR. Urinary tract infection may arise as a complication of poor bladder emptying secondary to BOO. A full assessment of voiding function is required when a man presents with AUR and UTI.

### Medications and AUR

Specifically, cholinergic antagonists and alpha-adrenergic agonists may precipitate urinary retention by inhibiting detrusor contractility and enhancing bladder outlet resistance, respectively. Medications administered for depression, allergies, Parkinson's disease and overactive bladder have anticholinergic properties. Alpha-agonists are a common component of over-the-counter cold remedies. A study by Athanasoupolos *et al*. demonstrated that tolterodine could safely be taken in combination with an alpha blocker in patients with urodynamically proven BOO, without the development of AUR.[19] Reynard *et al*. have demonstrated that anticholinergic use in BPH patients leads to a small increase in the post-void residual (PVR) without significantly increasing the risk of AUR.[20] Both these agents can be safely combined in the management of BPH, especially in patients with predominantly irritative symptoms and low PVR. When AUR does develop in association with BPH and concomitant use of these medications, management should follow standard procedure.

### Non-steroidal anti-inflammatory drugs (NSAIDS) and AUR

A population-based prospective case-control study of men >45 years to examine the role of NSAID use in AUR in the Netherlands documented that compared with nonusers of NSAIDs current users of NSAIDs had a 2.02-fold higher risk of AUR. Patients who recently started taking NSAIDs at a dose equal to or higher than the recommended daily dose had the highest risk for AUR (adjusted odds ratio [OR], 3.3). Past use of NSAIDs was not associated with increased rate of AUR. NSAIDs are known to have a direct effect on prostaglandin synthesis (especially PGE2) which plays an important role in contractions of the detrusor muscle.[21]

## PREDICTORS OF AUR

The factors which are considered as predictors of AUR in BPH can be divided into baseline and dynamic variables. Baseline variables are age, severe LUTS, low peak urinary flow rate, increased post-void residual urine volume (PVR), enlarged prostate, high serum PSA levels and previous conservatively managed AUR. The dynamic variables are worsening of IPSS >4 points, bothersome score > 3 points during treatment, increasing PVR, no response to alpha blockers. These dynamic variables can be actively monitored during the treatment period to predict the occurrence of AUR and assess the need for BPH surgery.[22]

## “SPONTANEOUS” VS. ‘PRECIPITATED” AUR

When dealing with a case of AUR in adult men, the use of the terms ‘precipitated’ or ‘spontaneous’ may be confusing if not qualified properly. It is very important to assess whether or not BPH is implicated in the etiology. In the vast majority of cases, AUR appears simply related to the natural history of BPH. If BPH is thought to be present, then the management is likely to involve a trial without catheter following administration of an alpha blocker. The presence of a factor that is considered to have precipitated or provoked the episode of AUR may influence the outcome in the longer term but there is little evidence that it alters the likelihood of a successful trial of voiding. Consequently, spontaneous and precipitated AUR may be considered to be the same when they are BPH-related, as the approach to initial management need not be altered.[23]

## MANAGEMENT OF AUR IN BPH

There is high variability among countries in real-life practice, in terms of duration of catheterization, hospital admission, management after a failed trial without catheter (TWOC) and emergency or delayed surgery.

### Suprapubic catheter vs. perurethral catheter

In a survey of 410 consultant urologists in the UK, urethral catheterization was used most often (98% of cases), with suprapubic catheter (SPC) only being used in failures for the management of AUR.[24] In another cross-sectional French survey including 2,618 men, the initial management consisted of urethral and suprapubic catheterization in 83% and 17% respectively.[25] Urethral catheterization is a technically easy procedure, less morbid compared to SPC and it can be done by community physicians. But it is associated with a higher rate of urine leak and injury to the urethra and bladder neck. Using an unrandomized design, Horgan *et al*. compared the outcome for three years after catheterization (suprapubic or urethral) in 86 consecutive patients. Of the 30 patients catheterized perurethrally, 40% had a UTI (vs. 10 out of 56 patients with SPC, 18%). Seventeen per cent of the patients catheterized urethrally developed urethral strictures, compared with none in the SPC group. A TWOC in seven of 11 patients who had been catheterized urethrally proved unsuccessful. Suprapubic catheterization was associated with less UTI (18% vs. 40%), less urethral stricture (0% vs. 17%), avoided urethral and bladder neck damage. Another advantage of the SPC is that the catheter can be clamped rather than removed while undergoing a TWOC, which avoids re-catheterization in case of failures. But SPC resulted in higher pain, hematuria and blockage of catheter.[26] The complications associated with SPC use are tube dislodgement, bowel perforation, acute peritonitis, urine extravasations, late bladder perforation and implantation of metastasis (in carcinoma bladder).

### Indwelling catheter vs. clean intermittent self-catheterization

In a comparative trial between indwelling catheter (IDC) vs. clean intermittent self-catheterization (CISC), the CISC group had a higher rate of spontaneous voiding than the IDC group (56% vs. 25%). The incidence of UTI was 32% in the CISC and 75% in the IDC group. After TURP, 20% in the CISC group had a UTI, compared with 69% in the IDC group.[27] The major advantage of CISC over IDC is the convenience of not having an external device and the maintenance of sexual activity. This study also showed that about half of the patients using CISC will eventually void spontaneously.

### Duration of catheterization

Djavan *et al* randomized men with AUR into three groups: in-and-out catheterization and dependent catheter drainage for two or seven days. On catheter removal, 44%, 51% and 62%, respectively, voided successfully. Patients who had retention volumes of >1300 mL benefited most from prolonged catheterization.[28] But prolonged catheterization may lead to increased incidences of urinary tract infection.

### Hospitalize vs. home with catheter

After catheterization, patients may be hospitalized or sent home and reviewed in the outpatient clinic. Country-specific differences in the percentage of patients hospitalized for AUR were found in a ‘real-life’ practice study conducted in various parts of the world. Most men presenting with AUR were hospitalized in France (69%) and Russia (80%), whereas few were admitted to the hospital in Mexico (22%), Denmark (25%) or the Netherlands (27%).[4] In the recent UK survey on the management of AUR, most urologists (65.5%) preferred to admit their patients after catheterization, while a further 19.3% would admit only if renal function was impaired. Only a minority (9.1%) would send the patient home with a catheter. Men hospitalized as a result of AUR stayed a mean of 5.0 days longer than men who were catheterized and sent home. Men who were admitted with AUR were more likely to require a second procedure for bleeding (4.6% vs. 1.7%). Complicated urinary infection was more common after surgery in men who were catheterized and sent home (15.6% vs. 9.5%) and consequently, more men in this group received antimicrobial agents after surgery (53.7% vs. 45.9%).[29] Prolonged catheterization leads to bacterial colonization of the urinary tract and might increase the risk of sepsis. However, no increased risk of major infective complications was detected. It is safe for a man with AUR to be catheterized and sent home to await an elective prostatectomy in the next few weeks. But admission is mandatory in case of renal failure, uro-sepsis, patients with severe comorbidity and patients who are difficult to follow.

### Trial without catheter

In the UK survey, 73.9% of men catheterized for AUR had a trial without catheter (TWOC), usually after two days of catheterization, while only 2.9% had immediate surgery. With failure of TWOC, 68.7% were re-catheterized with delayed surgery and 11.7% had a subsequent further TWOC later. In the French survey also, TWOC was standard, being used in 72.8% of cases after a median of three days of catheterization. If the TWOC failed most men (57.5%) were re-catheterized and had elective surgery. Some factors influence the success of a TWOC; lower age (< 65 years), high detrusor pressure (> 35 cmH_2_O), a drained volume of < 1L at catheterization, an identified precipitating factor (e.g., postoperative AUR) and prolonged catheterization are usually associated with a greater success rate of TWOC. Nevertheless, catheterization for > three days is associated with significantly higher comorbidity (hematuria, urosepsis and urinary leakage around the catheter) and double the rate of prolonged hospitalization than in men catheterized for < three days. There is increasing evidence that immediate treatment by bladder decompression can effectively be followed by a TWOC, which involves removing the catheter after one to three days, allowing the patient to void successfully in 23-40% of cases and surgery, if needed, to be performed later.

### Role of alpha blockers

Acute urinary retention related to BPH may be consecutive to a sudden stimulation of alpha 1 - adrenergic receptors. The rationale for the use of alpha blockers in BPH is that it acts on the “dynamic” component of bladder outlet obstruction by relaxing smooth muscle fibers located in the prostate and its capsule, bladder neck and prostatic urethra.[30]

The role of alpha blockers in the primary prevention of AUR in symptomatic BPH patients has been well studied. In a six-month placebo-controlled study involving 518 patients IR-alfuzosin was associated with a significantly lower incidence of AUR than placebo (0.4% vs. 2.6%). Among 3228 patients treated with IR-alfuzosin for three years, the incidence of AUR with alfuzosin was only 0.3% vs. 2-3% in the watchful waiting group. Emberton M *et al* analyzed the incidence of AUR in 5,792 men with BPH receiving alfuzosin at 10 mg once daily for six months. The study population included 3.8% patients with prior history of AUR managed conservatively. The rate of AUR and BPH-related surgery during treatment was 0.5% and 1.1% respectively.[11]

Several trials have been conducted assessing the ability of alpha blockers to improve the outcomes of TWOC (secondary prevention of AUR). McNeill *et al* showed that alfuzosin, when administered for two consecutive days in patients with AUR, was associated with a significantly higher rate of successful voiding after removing a catheter (on the second morning of treatment) than was a placebo (55% vs. 29%).[31] In the Alfuzosin in Acute Urinary Retention (ALFAUR) study, a large randomized, double-blind, placebo-controlled study which enrolled 360 patients with a first episode of AUR related to BPH, alfuzosin 10mg once daily significantly increased the rate of success of a TWOC, compared with placebo (62% vs. 48%). The multivariate analysis accounting for all confounding factors confirmed that alfuzosin almost doubled the likelihood of a successful TWOC.[5] The benefit of alfuzosin in facilitating a return to normal voiding was further confirmed in the French survey on the management of AUR. Of the 1906 men who had a TWOC, 1505 (79%) received alpha 1 -adrenergic blocker at the time of catheter removal (alfuzosin 76%, tamsulosin 6%, unspecified 18%). The TWOC success rate was significantly higher in those men receiving an alpha-1 -blocker (53.0% vs. 39.6% with out alpha-1-blocker). In the second phase of the ALFAUR study 165 patients with successful voiding were re-randomized to receive alfuzosin 10mg once daily or placebo for six months. Within the six-month follow-up 24.1% of the placebo-treated patients and 17.1% of alfuzosin-treated patients required BPH surgery, mainly for recurrent AUR. Hence the role of alpha blockers in both the primary and secondary prevention of AUR in BPH is well established.

### Role of 5-alpha reductase inhibitors in AUR

5-alpha reductase inhibitors reduce the prostate size by acting on the epithelial component of the BPH. In a double-blind, randomized, placebo-controlled trial which studied 3040 men with moderate to severe LUTS and enlarged prostate glands who were treated daily with 5 mg of finasteride or placebo for four years, AUR developed in 99 men (7%) in the placebo group and 42 men (3%) in the finasteride group. There was 57% reduction in risk with finasteride.[6] In a large retrospective comparative analysis Muta M *et al*. compared the rates of AUR and prostate surgery in BPH patients treated with the two currently available 5ARIs, dutasteride and finasteride. After controlling for background covariates, dutasteride-treated patients were 49.1% less likely to experience AUR than patients treated with finasteride (*P*=.0207). Patients treated with dutasteride were also less likely to undergo prostate-related surgery, (1.4% of dutasteride-treated patients and 3.4% of finasteride-treated patients).[32]

### Combined medical management

The MTOPS trial is the largest and longest clinical study of medical therapy for BPH to date. A total of 3047 men were randomized to receive placebo, finasteride, doxazosin or combination therapy for a mean of 4.5 years. The reductions in risk of AUR in the finasteride, doxazosin and combination groups were 68%, 35% and 81%, respectively. The risk reductions for surgical intervention in the finasteride, doxazosin and combination groups were 64%, 34% and 67%, respectively. The MTOPS study hence confirmed that combined medical treatment significantly reduces the risk of progression to AUR and the need for surgical intervention in BPH patients. Kim CI *et al*., from a retrospective analysis of 341 patients (192 patients in alpha blocker only group, 149 patients in combination treatment, followed up for six to eight years) reported the incidence of AUR in BPH patients receiving alpha blockers alone and combination of alpha blocker and a-alpha- reductase inhibitor. In the alpha blocker group 17.7% patients and 12.1% in the combination group experienced AUR (*P* < 0.05).[33]

### Surgical management

Acute urinary retention was once considered an absolute indication for prostatectomy. A report by the National Prostatectomy Audit Steering Group offers a unique look at the outcomes of prostate surgery in men with AUR. In five UK healthcare regions, 3966 prostatectomies were performed in 56 hospitals by 103 surgeons. The 1242 patients presenting with AUR were older and had larger glands and more comorbidities. The patients with AUR had an excess risk of death at 30 days (RR 26.6; 3.5-204.5 CI) and at 90 days after surgery (RR 4.4; 2.5-7.6 CI). In addition, the risk of intra- and perioperative complications was higher in the AUR group. It has also been found that immediate prostate surgery is associated with increased perioperative complications than delayed surgery in patients presenting with AUR. But bacterial colonization of a urinary catheter is significantly greater after three days of catheterization and can result in major morbid events such as fever and possible progression to bacteremia. Hence in BPH patients presenting with AUR, it is essential to delay the surgical treatment by initially offering TWOC following the use of alpha blockers.

### Role of pressure-flow studies

Some patients with AUR due to BPH do not have successful outcome after prostatectomy and require either a chronic indwelling urethral catheter or clean intermittent catheterization. To investigate the utility of “late” pressure-flow studies in predicting the outcome of prostatectomy for AUR, Dubey *et al*. prospectively assessed 58 patients with AUR using the International Prostate Symptom Score and pressure-flow studies at a median (range) of 24 (13-60) days after the episode of retention and before transurethral resection of the prostate. It was found from the study that absence of bladder instability, inability to void during the pressure-flow study and a maximal detrusor pressure of < 20 cmH_2_O are associated with a poor outcome after prostatectomy. Hence in patients with AUR, pressure-flow studies undertaken after a period of adequate bladder rest are useful in predicting the surgical outcome.[34]

## NEWER THERAPIES IN THE MANAGEMENT OF AUR

### Endothelin antagonists

Human prostate tissue produces endothelin and in the presence of smooth muscle, contractions have been observed which are not inhibited by a-blockers. Recently, a new orally active endothelin antagonist was found to inhibit endothelin-induced prostatic contractions in a dose-dependent way.[35]

### Prostatic devices/stents

Patients with AUR are often elderly men with several coexisting diseases, which multiply the risks of surgery. Alternative modes of treatment for AUR thus need to be studied. A thermosensitive nitinol (Memokath H) urethral stent was used in the study reported by Kiyota *et al*., to treat 17 patients with BPE, 15 of whom had AUR. The mean peak urinary flow rate increased from 2.0 mL/s before placement to 12.9 mL/s afterward, during a mean follow-up of seven months. Combined therapy comprising a bioabsorbable self-reinforced poly L-lactic acid (SRPLLA) urethral stent and finasteride was studied in 11 men with AUR who were treated as outpatients. All of them had a suprapubic catheter inserted and the SR-PLLA stent was placed cystoscopically. The patients were allowed to attempt to void spontaneously after two days. All patients voided spontaneously within two weeks. There was a steady improvement in urinary flow rates up to nine months, followed by a slight impairment after the bioabsorption of the stent. During the mean (range) follow-up of 24 (23±26) months only three patients required surgical treatment. The bioabsorbable stent keep the bladder outlet open till the time finasteride starts to reduce the size of the prostate.[36]
